# Effect of clinical decision support systems on emergency medicine physicians' decision-making: A pilot scenario-based simulation study

**DOI:** 10.3389/fped.2022.1047202

**Published:** 2022-12-15

**Authors:** Azadeh Assadi, Peter C. Laussen, Gabrielle Freire, Marzyeh Ghassemi, Patricia C. Trbovich

**Affiliations:** ^1^ Labatt Family Heart Centre, Department of Critical Care Medicine, Hospital for Sick Children, Toronto, ON, Canada; ^2^ HumanEra, Institute of Biomaterials and Biomedical Engineering, Department of Engineering and Applied Sciences, University of Toronto, Toronto, ON, Canada; ^3^ Institute of Medical Sciences, University of Toronto, Toronto, ON, Canada; ^4^ Executive Vice President for Health Affairs, Boston Children’s Hospital, Boston, MA, United States; ^5^ Professor of Anaesthesia, Harvard Medical School, Boston, MA, United States; ^6^ Division of Emergency Medicine, Department of Pediatrics, University of Toronto, Toronto, ON, Canada; ^7^ Electrical Engineering and Computer Science, Massachusetts Institute of Technology, Boston, MA, United States; ^8^ Institute for Medical Engineering & Science, Massachusetts Institute of Technology, Boston, MA, United States; ^9^ Vector Institute, Toronto, ON, Canada; ^10^ CIFAR AI Chair, Vector Institute, Toronto, ON, Canada; ^11^ Institute of Health Policy Management and Evaluation, University of Toronto, Toronto, ON, Canada; ^12^ Research and Innovation, North York General Hospital, Toronto, ON, Canada

**Keywords:** clinical decision support (CDSS), congenital heart disease, pediatric, emergency medicine, decision making, digital health, macrocognition

## Abstract

**Background and objectives:**

Children with congenital heart disease (CHD) are predisposed to rapid deterioration in the face of common childhood illnesses. When they present to their local emergency departments (ED) with acute illness, rapid and accurate diagnosis and treatment is crucial to recovery and survival. Previous studies have shown that ED physicians are uncomfortable caring for patients with CHD and there is a lack of actionable guidance to aid in their decision making. To support ED physicians' key decision components (sensemaking, anticipation, and managing complexity) when managing CHD patients, a Clinical Decision Support System (CDSS) was previously designed. This pilot study evaluates the effect of this CDSS on ED physicians' decision making compared to usual care without clinical decision support.

**Methods:**

In a pilot scenario-based simulation study with repeated measures, ED physicians managed mock CHD patients with and without the CDSS. We compared ED physicians' CHD-specific and general decision-making processes (e.g., recognizing sepsis, starting antibiotics, and managing symptoms) with and without the use of CDSS. The frequency of participants' utterances related to each key decision components of *sensemaking, anticipation,* and *managing complexity* were coded and statistically analyzed for significance.

**Results:**

Across all decision-making components, the CDSS significantly increased ED physicians' frequency of “CHD specific utterances” (Mean = 5.43, 95%CI: 3.7–7.2) compared to the without CDSS condition (Mean = 2.05, 95%CI: 0.3–3.8) whereas there was no significant difference in frequencies of “general utterances” when using CDSS (Mean = 4.62, 95%CI: 3.1–6.1) compared to without CDSS (Mean = 5.14 95%CI: 4.4–5.9).

**Conclusion:**

A CDSS that integrates key decision-making components (sensemaking, anticipation, and managing complexity) can trigger and enrich communication between clinicians and enhance the clinical management of CHD patients. For patients with complex and subspecialized diseases such as CHD, a well-designed CDSS can become part of a multifaceted solution that includes knowledge translation, broader communication around interpretation of information, and access to additional expertise to support CHD specific decision-making.

## Introduction

Children born with congenital heart disease (CHD) are predisposed to rapid clinical deterioration when they become acutely ill with common childhood illnesses compared to children without CHD (1). CHD refers to the range of anatomic defects of the heart and its great vessels that effect the flow of blood through the heart and the lungs. The increased hemodynamic fragility of these patients stems from the natural history of their underlying CHD (despite corrective interventions) and the burden of residual lesions which also alter the responses of these children to traditional resuscitative measures (1). During these episodes of acute illness, patients with CHD often present to their local emergency departments (ED) where specialized training in pediatric CHD is often limited. Of the 241 million ED visits recorded in United States' Nationwide Emergency Department Sample between 2006 and 2014, CHD patients represented 0.17% of these visits (2). The majority of these patients were under 1 year of age and were more likely to die, require hospital admission, or transfer to specialty centers compared to children without CHD (2).

The incidence of CHD is approximately 8 in 1,000 live births (3). The anatomic variations of CHD are many and complex; one group of patients who are particularly vulnerable and high risk are those born with only one main pumping chamber (ventricle). In this form of CHD, blood from the body and the lungs mixes in the heart creating an oxygen level (saturation) in the blood that is lower (usually <85%) when compared to a patient with two normal ventricles. When ED physicians in the state of Michigan were surveyed on their degree of comfort caring for acutely ill pediatric patients with a single ventricle, they expressed an overall lack of comfort (4). When asked about the expected oxygen saturation in these patients, 52% of general ED physicians and 35% of pediatric ED physicians were unsure of what the expected oxygen saturations should be (4). Moreover, 18% of general ED physicians and 26% of pediatric ED physicians identified the wrong saturations for these patients (4). Some reasons for this lack of comfort and familiarity with CHD patients among ED physicians include limited specific training in acute CHD, lack of available and detailed information about the CHD in a particular patient, the unique and complex medical language used by experts to describe CHDs, and limited access to in-house CHD experts (4–6).

**Figure 1:**
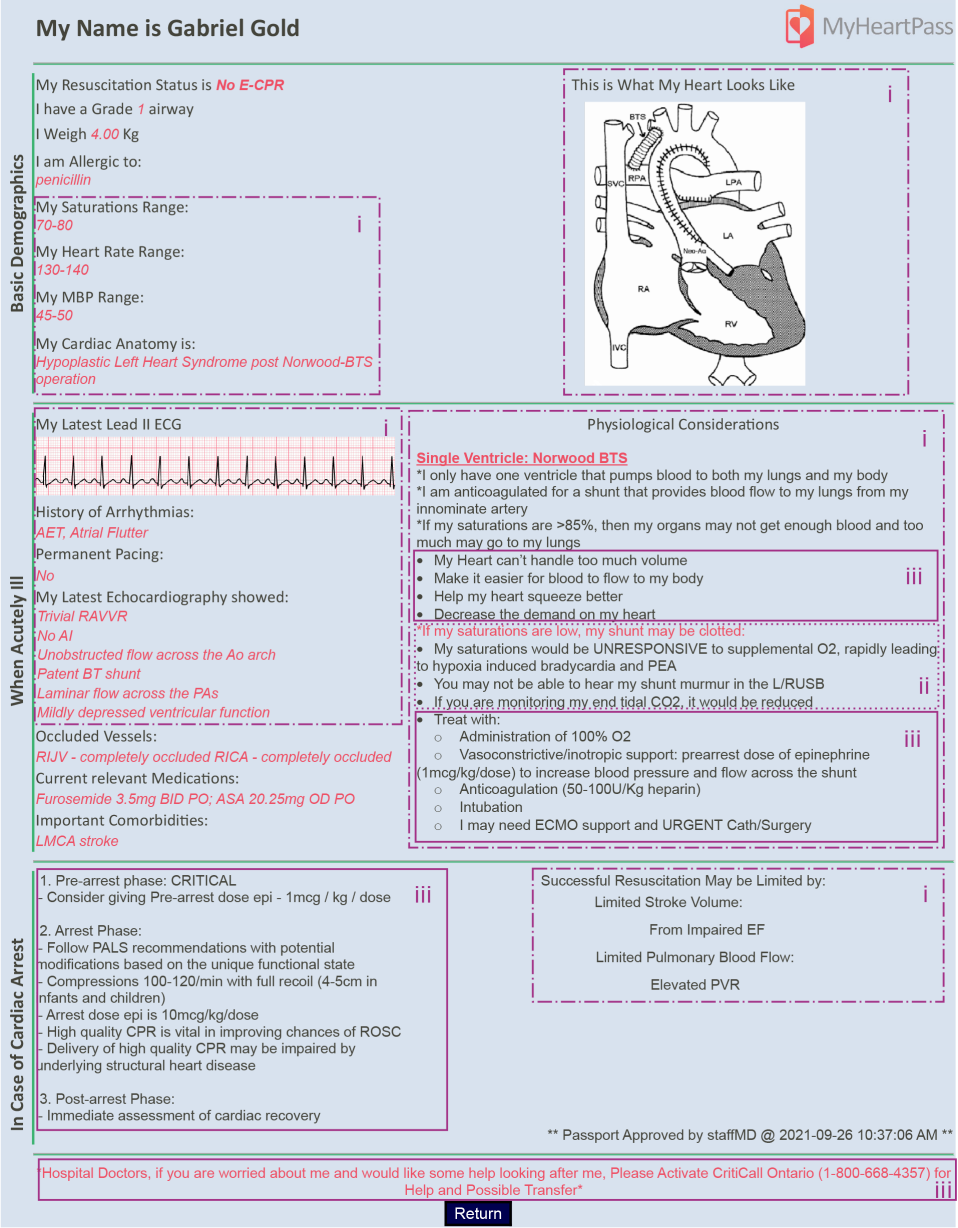
Screenshot of *MyHeartPass™* prototype. Sections marked with (i) refer to elements of design pertaining to key decision requirement *distinguish the patient's unique physiology based on their unique cardiac anatomy.* Elements marked with (ii) capture the elements pertaining to the key decision requirement of *explicitly consider CHD specific differential diagnoses to allow a more structured reflection of diagnosis;* and (iii) capture the elements pertaining to the key decision requirement of *select CHD appropriate interventions for each patient*.

We identified a lack of actionable guidance to support ED physicians in their decision making when confronted with a patient with CHD and intercurrent acute illness (7), and developed a prototype Clinical Decision Support System (CDSS) using the critical decision method (CDM), called *MyHeartPass^TM^* (Figure 1) (8). CDSS are systems that provide clinicians with disease and patient specific clinical knowledge and information to facilitate effective decision making, enhance patient care, and improve outcomes (9, 10). These systems have improved care of patients through a variety of means including drug calculations, making patient information more accessible and providing supplemental information to clinicians (10). Often, CDSS studies focus on evaluating outcomes following the use of a CDSS without showing how or why they fail or succeed in modifying outcomes or decision making (10–15). In this pilot study, we use a scenario-based simulation to analyze the frequency and typology of decisions made using the prototype CDSS compared to usual care without clinical decision support to assess which decision making components are modified.

## Previous work

We designed *MyHeartPass^TM^* using semi-structured interviews of CHD experts (pediatric cardiac intensivists) and ED physicians. The transcripts from CHD experts’ and ED physicians' retrospective recounts of challenging scenarios were used to compare their macrocognitive processes (16, 17). We identified and designed for the following key decision requirements that were least supported: (i) *Sensemaking – distinguishing patients’ unique physiology based on their cardiac anatomy,* (ii) *Anticipation – Considering CHD-specific differential diagnoses to allow a more structured reflection of diagnosis,* and (iii) *Managing Complexity – selecting CHD-appropriate therapeutic interventions* (7).

## Methods

Ethics approval was obtained from the Research Ethics Board (REB 1000064567) of a large academic institution. Before participating in the scenarios, all participants were asked brief demographic questions and their degree of comfort managing patients with a specific type of CHD (patients with single ventricle physiology) who are considered most fragile and most likely to acutely present to the ED. This pilot study was conducted as a partial counter-balanced repeated measures pilot study where half the participants completed their first scenario with the CDSS and the second without.

### Materials

Interviews were conducted using *Microsoft Teams®,* a virtual meeting platform, and *Miro* (Version 0.7.2), an individualized virtual idea board. In scenarios where participants had access to the CDSS, *MyHeartPass™* was used as the prototype CDSS for the scenario. Scenario data was provided to all participants based on the scenario they were in (Table 1). In keeping with current practice, for all scenarios, supplemental documents and diagnostic data were made available to participants only when they would ask for it (Table 1). These supplemental documents and diagnostic data are routinely available during clinical care but have to be deliberately accessed by clinicians managing the patient. All participants were presented with *MyHeartPass™* at the start of their CDSS Scenario with a brief 5-minute orientation to the CDSS.

**Table 1:** List of available data, documents, and diagnostic results made available to participants.

Scenario Data	Supplemental documents	Diagnostic Data
• History of presenting illness • Brief CHD history (name of the specific CHD and time since last surgical intervention) • Presenting vital signs • Presenting clinical exam	• Most recent office visit note • Most recent hospital discharge note • Most recent echocardiography report • List of current medications • List of current issues • Pre-discharge electrocardiogram • Pre-discharge Chest x-ray	• Vital sign data reflecting patients’ response to participants’ proposed interventions • Clinical exam data reflecting patients’ response to participants’ proposed interventions • Results of diagnostic tests prescribed by participants ○ Chest x-ray obtained during the scenario ○ Point of care ultrasound of the lungs/heart obtained during the scenario ○ Electrocardiogram obtained during the scenario ○ Blood test results • Advice from CHD expert when sought by participants

CHD, congenital heart disease.

### Setting and participants

As the intended end users of *MyHeartPass™*, pediatric emergency medicine physicians working in a quaternary pediatric hospital were recruited to participate in this pilot study. The ED in the pilot study hospital sees over 50,000 pediatric patients, has 6,000 admissions annually (18), and at the time was staffed with 26 full-time and part-time ED physicians. Of these physicians, 22 met the following inclusion criteria: (a) no formal training in acute pediatric CHD treatment, (b) at least 3 years of experience in the field of pediatric emergency medicine, and (c) actively practicing as staff physicians at the time of the pilot study. The pilot study hospital was a major cardiac center with an average of 550 pediatric cardiac surgeries per year. In addition to caring for these patients post-operatively, the cardiac intensive care unit of this hospital also medically managed pediatric congenital and acquired heart disease patients (19). Based on their training, experience, and role in managing these patients at their highest acuity, pediatric cardiac intensivists were deemed to be experts in the acute management of pediatric patients with CHD. The pediatric cardiac critical care unit of the pilot study hospital was staffed with 9 full-time physicians.

### Virtual simulations and scenarios

All virtual simulations were conducted one-on-one on *Microsoft Teams®*. Participants were free to choose a location of their convenience for this simulation (i.e., their personal office at work or at home). Simulations were run by AA, a pediatric nurse practitioner in the cardiac intensive care unit at the pilot study institution and a doctoral student in human factors engineering with experience conducting medical scenario-based simulations and research interviews. At the onset of the pilot study, participants were briefed on the steps of the pilot study and broadly oriented to *MyHeartPass™* and *Miro*. Given that participants had already used *Microsoft Teams®* and were familiar with the platform, no orientation to this virtual meeting platform was provided. Participants were also informed that for all scenarios, they can ask for any additional information (i.e., items from the supplemental documents and diagnostic data in Table 1) that they normally would, and those results will be made available to them as they would be under the current standard of care conditions.

Mock scenarios were specifically designed by CHD experts (AA and PL) from the study team for this virtual simulation pilot study. The scenarios resembled the presentation and clinical evolution of acutely ill pediatric patients with CHD in an ED. Specifically, an intercurrent illness was incorporated into all the scenarios and each patient was made to deteriorate based on one of their common CHD specific mechanisms of deterioration. Each scenario was presented in stages, allowing participants to provide their differential diagnoses, thoughts, rational, and actions at each stage. These responses were documented on *Miro*. Scenarios were stopped when participants identified the right diagnosis and treatment recommendations, after obtaining a complete consultation from cardiology, or after reaching the allotted time, whichever came first. For member checking, each participant was asked to review their actions and decisions on *Miro* at the culmination of each scenario to ensure nothing was omitted or misinterpreted. Member checking is a validation technique that is used to explore the credibility of the results by seeking feedback from participants (20). A small pilot study of all the scenarios was completed with a CHD expert outside the study team in advance of participant recruitment to ensure scenarios were similar in complexity, clinically accurate and realistic while also ensuring the questions and the simulation itself ran smoothly within the allotted time.

ED physician participants completed two scenarios in total, one with *MyHeartPass™* and another without. Each scenario was allotted 30 min to reflect the approximate amount of time these patients would spend in the ED before being seen by cardiology or the rapid response team from intensive care. CHD-expert participants completed all their scenarios without *MyHeartPass™* within the same allotted time as the ED physicians.

### Data analysis

Scenarios were audio and video recorded, transcribed verbatim, and analyzed using NVivo 12® software. Two coders (AA and TR) with extensive experience coding interview data analyzed the interview transcripts. Coders independently identified utterances of decisions and reviewed their decision utterances for overlap (i.e., did they both identify the same decision utterances) to come to consensus. Once all utterances of decisions were identified, coders independently coded each decision utterance for key decision components based on *a priori* operational definitions identified in a previous study (Table 2) (8). They also coded based on whether a decision utterance was CHD-specific (e.g., choosing to start an epinephrine infusion to support the function of the heart and increase cardiac output) or a general decision component (e.g., choosing to start antibiotics to treat a potential infection) to evaluate the effect of the CDSS on participants' non-CHD specific (general) decision making (i.e., recognition, investigation, and management of the intercurrent illness and other possible differential diagnoses). Only those decision utterances that were relevant to understanding patient state, diagnosing, and evaluating hypotheses as well as the management of the patient in the scenario were included in the analysis (e.g., prescribing an epinephrine infusion to support patients heart function [relevant] vs. describing how many times they have prescribed epinephrine for these patients in the ED [irrelevant]). The range of relevant utterances was defined based on the range of utterances of CHD experts and the clinical expertise of AA (also a CHD expert). Repeated utterances of the same decision were only counted once (e.g., if a participant asked for an ECG twice without describing a new indication for a repeat ECG, only their first utterance of the decision was counted). Researchers compared coding for inter-rater reliability until *k* > 0.7 was achieved (21), at which point, a single researcher proceeded to code all subsequent simulations. All coded simulations were charted in a matrix. The matrix consisted of a table that organized the utterances by the simulation in which they occurred, and by the key decision components (CHD-specific vs. general). This matrix summarized the utterances across the key components which facilitated analysis of their frequency and qualitative characteristics. Frequency of utterances were analyzed in a 3 (decision making component: sensemaking vs. anticipation vs. managing complexity) × 2 (format: without CDSS vs. with CDSS) × 2 (type: general non-cardiac vs. CHD-specific) repeated measures analysis of variance (ANOVA). Analyses were conducted using IBM SPSS Version 26 (IBM Corp., 2014), *α* < 0.05 with Bonferroni correction for pairwise comparisons.

**Table 2:** Analytical framework.

Codes		Definitions
CHD-specific Decision Components	Sensemaking	*Understanding patient state:* understanding patients’ **cardiac anatomy** (structure of the heart and its great vessels as well as the pattern of blood flow through it), the resulting **physiology** (describe the physiology and its implications for patient and treatment), and the associated **acceptable baseline Vital Signs** (e.g., oxygen saturation, heart rate, blood pressure, electrocardiogram, etc.). Understanding patients’ risk state based on their anatomy and physiology.
	Anticipation	*Determine potential diagnoses:* Identify possible **differential diagnoses** and associated mechanism of deterioration based on patients’ unique CHD. Identify **diagnostic tests** (e.g., obtaining echocardiography to evaluate shunt patency, observing for specific responses to treatments, etc.) to narrow possible diagnoses and hypotheses.
	Managing Complexity	*Therapeutic interventions:* Initiating **interventions to treat the cardiac physiology/pathology** and to maintain or restore hemodynamic stability and mitigate risk. (e.g., starting inotrope and vasopressors, administering fluids to optimize cardiac physiology, etc.)
General Decision Components	Sensemaking	*Understanding patient state:* recognize signs and symptoms that are abnormal and concerning in pediatric patients and their association with various disease states.
	Anticipation	*Determine potential diagnoses:* Identify possible non-cardiac **differential diagnoses** and mechanism of deterioration based on the presenting symptoms and patient history. Identify **diagnostic tests** (e.g., obtaining blood cultures, point of care ultrasound to identify pleural effusion vs. lung consolidation, etc.) to narrow possible diagnoses and hypotheses.
	Managing Complexity	*Therapeutic interventions:* Initiating **interventions** to treat the patient and their illness (e.g., obtaining intravenous access, administering antibiotics, providing respiratory support, etc.). These interventions do NOT target a specific cardiac physiology.

## Results

### Overview

In total, 7 of the eligible 22 pediatric ED physicians (32%) participated in this pilot study, all of whom were able to complete their scenarios within the allotted time. Table 3 shows demographic characteristics of the ED participants. Three of the 9 CHD experts (33%) also participated in the pilot study as study controls.

**Table 3:** Ed physician participant characteristics.

		Number of Physicians
Participants’ Gender	Female	4
	Male	3
Experience within their specialty	More than 15 years	1
	5–15 years	4
	Less than 5 years	2
Experience with acute CHD treatment	More than 30 patients/year	1
	10–30 patients/year	5
	<10 patients/year	1
Comfort treating a pediatric single ventricle without support from in-house pediatric cardiology	Very comfortable	0
	Comfortable	1
	Somewhat Uncomfortable	3
	Uncomfortable	3
	Worried	0

**Figure 2:**
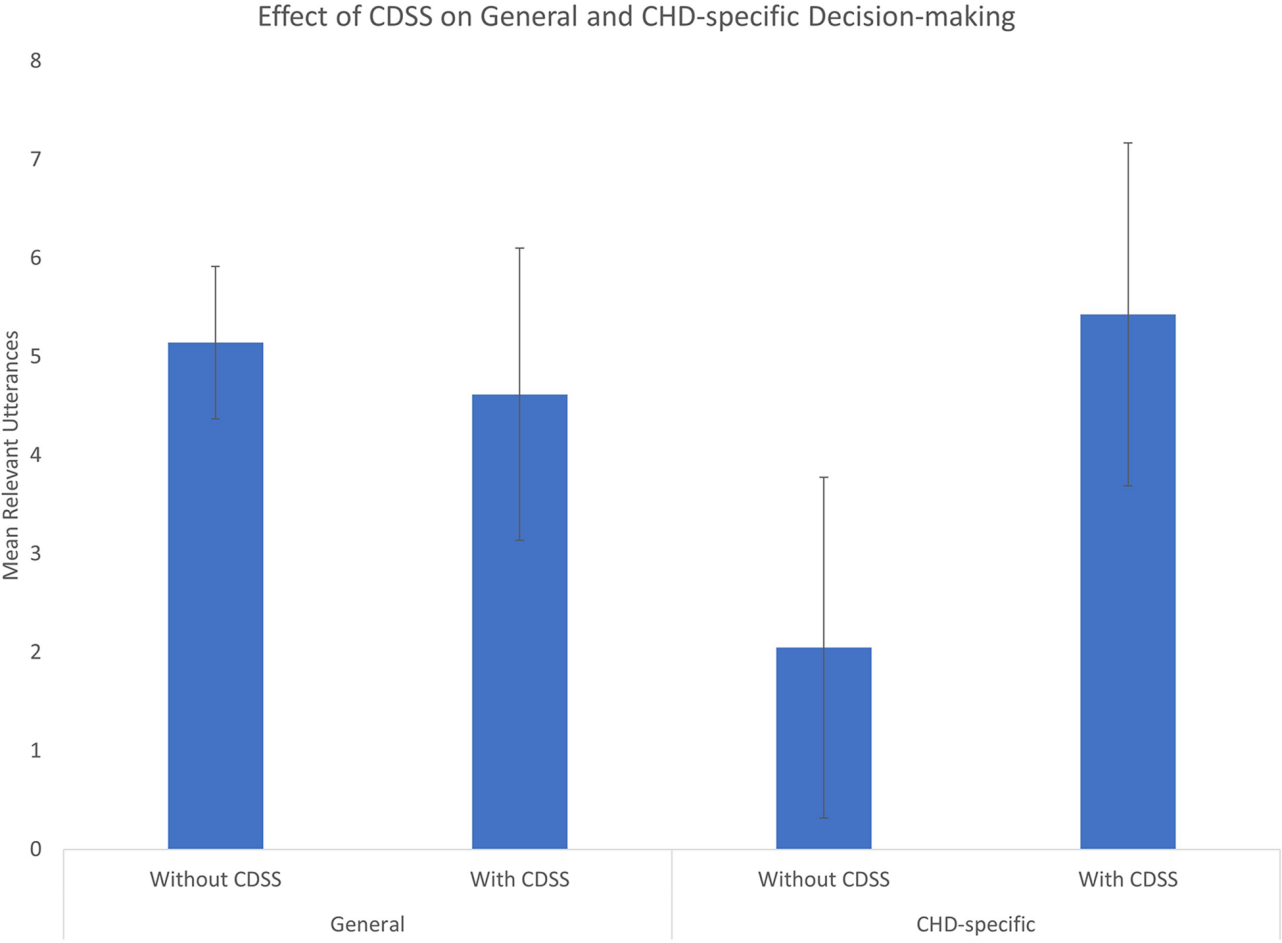
Effect of *MyHeartPass™* on ED physicians**’** general and CHD-specific decision-making. The use of *MyHeartPass™ (CDSS)* is seen to have increased utterances of CHD-specific decisions without effecting utterances of general decisions. Error bars represent the 95% confidence interval.

Although the three-way interaction was not significant, there was a significant interaction between format (i.e., with and without CDSS conditions) and type (general and CHD-specific utterances). Specifically, across all decision-making components, the CDSS significantly increased ED physicians' frequency of “CHD-specific utterances” (Mean = 5.429) compared to the without CDSS condition (Mean = 2.048) whereas there was no significant difference in frequencies of “general utterances” when using CDSS (Mean = 4.619) compared to without CDSS (Mean = 5.143) [*F*(1,6) = 24.48, *p* < 0.01] (Figure 2).

### Effect of CDSS on each CHD-specific decision components

A detailed review of ED physicians' utterances of decisions reveals the ways in which *MyHeartPass™* effects each of their 3 key decision components.

#### Sensemaking – understanding patient's CHD-specific state

ED physicians always referred to the patients' acceptable baseline oxygen saturations in *MyHeartPass™* to not only understand what oxygen saturation they should target for the patient when choosing treatments, but also to understand their risk state. For example, during the blocked Blalock-Taussig (BT) shunt scenario, PEM02 referenced *MyHeartPass™,* commenting that “*here (baseline saturation is) listed between 70 and 80. (It) is actually super helpful because 64 is not as awful as it could be if their saturations were supposed to be at 85 to 95 for example.”* Conversely, in the absence of *MyHeartPass™,* ED physicians treated CHD patients based on what they assumed would be appropriate saturations for the patient until confirming it with consulting CHD experts or the patients' electronic health records.

ED physicians also used the graphical illustration of patients' unique cardiac anatomy and associated description of physiological states in *MyHeartPass™* to conceptualize the anatomy and begin to understand the effect of anatomy on patient physiology. For example, PEM04 explained that in patients with Fontan physiology “*flow to the lungs is passive and there*”*s this huge sort of restriction to that because of the pathology in the right lung.”* Without *MyHeartPass™* however, they did not describe the patients' cardiac physiology or risk state as part of their patient assessment.

#### Anticipation – formulating CHD-specific differential diagnoses and testing hypotheses

After an initial phase of *sensemaking* and establishing an understanding of the patient's current state, participants engaged with the decision component of *anticipation* and formulated differential diagnoses and prescribed diagnostic tests to narrow their list of differentials and test their hypotheses. This was an iterative process where new data modified clinicians' *sensemaking* which in turn modified their list of possible differential diagnoses. While the use of *MyHeartPass ™* improved ED physicians' *sensemaking,* it was not effective at bridging the gap between identifying heart failure as a possible diagnosis and the specific cardiac cause to explain the failure. For example, when treating the patient with the failing Fontan circulation, PEM07, like most other participants, was able to identify that the patient was in “*a septic shock or a cardiogenic shock… the two most likely things and they could be both happening.”* However, despite correctly diagnosing the pleural effusion (a non-cardiac diagnosis) on x-ray and then on point of care ultrasound, they were unable to make the link between the pleural effusion and the failure of the patient's Fontan circulation.

Nevertheless, with *MyHeartPass™,* ED physicians correctly prescribed more physiology based diagnostic investigations (e.g., echocardiography to assess patency of shunts, point of care ultrasound to evaluate cardiac function, and apply oxygen to determine cause for desaturation in certain heart disease), particularly when the test was recommended in the CDSS. For example, when treating the patient with obstructed BT shunt using *MyHeartPass™,* PEM06 was able to identify a lack of response to supplemental oxygen as a positive diagnostic indicator for “*a blocked BT shunt”* and proceed to request an echocardiography to confirm this suspicion. This contrasted with when ED physicians did not have *MyHeartPass™,* they prescribed fewer physiology specific diagnostic tests. For example, they broadly asked to speak with the CHD expert, expecting them to determine the specific questions to ask on echocardiography and the urgency with which the test was required.

#### Managing complexity – treating the patient with CHD-specific interventions

Participants initiated interventions at various points throughout each scenario. Some interventions were delivered to treat the underlying physiology, such as initiating epinephrine to support the function of the heart or starting positive pressure to offset work of breathing and oxygen consumption. These physiology-based interventions occurred more frequently when ED physicians had access to *MyHeartPass™.* For example, when PEM03 read the latest echocardiography of the mock patient in *MyHeartPass™* said “*I would consider starting her on presser like Epinephrine infusion to help with her myocardial demand. Looking at her latest Echo show some decrease systolic function and it says here that she does have systolic dysfunction at baseline, so my concern is … I need her to pump to increase to optimize her cardiac output”.* However, in the absence of *MyHeartPass™,* ED physicians often focused on treating symptoms rather than the CHD physiology. For example, when treating the mock Fontan patient with Fontan failure due to poor pulmonary blood flow in the context of a large pleural effusion, PEM04 considered *“volume resuscitation vs. diuretics with regard to, you know, potentially needing to start Epinephrine”* without appreciating the effect of each intervention on the failing Fontan circuit.

In general, when ED physicians had access to *MyHeartPass™,* they readily opted to use inotropes and pressors (e.g., epinephrine) to support blood pressure and augment cardiac output. Without the use of *MyHeartPass™,* ED physicians were more hesitant to use these drugs, often opting to manage hemodynamics with cautious fluid administration instead.

### Effect of CDSS on decision making

The use of *MyHeartPass™* did not have any significant influence on ED physicians' general utterances of decision making. Importantly, the use of *MyHeartPass™* did not lead to any of the participants missing the key general differential diagnoses (i.e., pneumonia and sepsis) and their associated treatments (i.e., escalation in respiratory support and administration of antibiotics). A summary of these results can be found in Figure 2.

Lastly, participants described *MyHeartPass™* as an overall helpful tool that makes patient specific information more readily available to them. Participants using *MyHeartPass™* described feeling empowered to advocate for their patients and seek further expertise. For example, PEM06 explains that “*You get a real baseline understanding of like where the kid is anatomically. (…) you can speak to the cardiologists, and you could actually start to have a more targeted discussion around. Hey, I see that (…) this kid has this BT shunt and is sating 60. Is it possible there's a shunt blockage?*” PEM06 goes on to say that “*It'll empower us to advocate for the cardiology fellow to come and do the echo in the ED because that's really what we're doing when we're calling (cardiology). We're trying to figure out should the cardiology fellow come and see the kid immediately in the emergency department? (…) It's very empowering to know that you could you yourself as the Emerge physician can come to the conclusion that yes, I think the cardiology fellows should come down to the ED, and this is why. That's empowering.”* Similarly, PEM07 said that “*it was nice to have something to go by”* when reviewing the patient with the CHD expert.

## Discussion

Findings from this pilot study show that the use of a prototype CHD CDSS improves the CHD-specific decision-making of ED physicians, specifically their *sensemaking, anticipation, and management of complexity*. Our findings show that the use of our prototype CDSS facilitated CHD-specific *sensemaking* through a greater understanding of patients' unique CHD anatomy, physiology, and expected vital signs. Improvements in *anticipation* were due to a greater number of CHD-specific diagnostic tests being prescribed by ED physicians with minimal improvement in their ability to identify the appropriate CHD-specific diagnosis. Despite not consistently identifying the correct diagnosis, the use of the CDSS still improved *management of complexity* in these patients by increasing the number of appropriate CHD-specific interventions ED physicians prescribed. There was also no significant effect on clinicians' general (non-cardiac) *sensemaking, anticipation* and *management of complexity* while using the proposed CDSS. Our findings therefore suggest that designing CDSS with a focus on specific decision-making cognitive processes can improve decision making, communication, and confidence among clinicians caring for acutely ill pediatric patients with CHD.

Like the participants in Cashen et al.'s (2011) study, most ED physicians in our pilot study were overall uncomfortable or somewhat uncomfortable managing pediatric patients with CHD without support from pediatric cardiology. In their study, Cashen et al. (2011) found a mismatch between the experience and comfort of general and pediatric ED physicians with single ventricle physiology and the educational priority the acute management of these patients should take. While the total number of pediatric patients with single ventricle physiology presenting to the ED with cardiorespiratory issues is small compared to all other causes of pediatric presentation, these patients are often hemodynamically fragile and deteriorate rapidly leading to a high burden of morbidity and mortality (2). Given the vast knowledge burden expected of ED physicians, additional training in the management of pediatric CHD may be insufficient on its own and made more effective with the use of sociotechnical solutions such as our proposed CDSS that aims to support clinicians' decision making. While not specifically measured, CDSS such as the one proposed here can also provide some reassurance and structure in clinicians' decision making as well.

To explain clinical and diagnostic reasoning, multiple theoretical constructs have been created. In general, they involve collection of clinical data utilized for the formulation of differential diagnoses based on clinicians' inherent knowledge and pattern recognition, and scientific reasoning (22). This is the process of sensemaking or understanding a patient's state. To refine the list of differentials, clinicians prescribe diagnostic tests to gather additional data and apply their broader knowledge of disease probabilities and other patient specific modifiers to ultimately decide on a diagnosis which would drive management decisions (22). Our findings show that the proposed CDSS was effective at augmenting CHD-specific decision making without distracting ED physicians from diagnosing and treating other non-CHD causes of illness based on their presentation. This is particularly important given that treating the intercurrent illness is part of the treatment strategy to improve the cardiac output in children who also develop heart failure given their CHD. The qualitative differences between ED physicians' *sensemaking, anticipation,* and *management of complexity* suggest the CDSS will not serve as a replacement to involving CHD experts in the management of these patients. Indeed, the ability to transform the fundamental understanding of a patient's CHD to what could be expected of their physiology in response to an intercurrent illness, specific diagnostic investigations to determine the illness physiology, and the rationale for various treatment choices requires expertise and experience in this area that cannot be replaced with a CDSS. Improving clinicians' *sensemaking* through the use of CDSS can, however, empower clinicians to have more informed discussions and dialogue with CHD experts, and approach the management of these patients from a primary cardiac perspective while concurrently managing underlying illnesses. This is particularly relevant with CDSSs that make prescriptive recommendations without triggering discussions to ensure relevance of recommendation in the context of the patient. To further support ED physicians' decision making, future designs of this tool should focus on augmenting *anticipation* through addressing the synthesis gap between *sensemaking* and *anticipation.* This involves a greater understanding of the CHD, the implication of patients' physiology, the natural history of the CHD, and the effect of residual lesions on possible causes of heart failure.

## Limitations

This pilot study was conducted as a single center pilot study at a major cardiac center, where the existing referral structures and greater CHD exposure of ED physicians may have influenced the findings of this pilot study and made the sample size small. To better understand the effect of CDSS on clinicians' decision making, the study should be expanded to evaluate the effect of variables such as participant role (i.e., trainees vs. nurses, vs. pediatric ED physicians vs. general ED physicians), scenario complexity (i.e., complex CHD vs. simple CHD), setting (i.e., community ED, vs. academic ED, vs. pediatric ED, vs. specialty centre ED), and gender. Despite the use of realistic scenarios, the simulated nature of this pilot study may have also affected participants' responses. However, as participants responses were compared against their own control scenarios, any simulation effect would be equal across both scenarios. The significant findings obtained in this pilot study, therefore, seem to be reflective of the intervention (i.e., CDSS) and not an artifact of simulation. Nevertheless, it is important to repeat this study in a near live simulation setting to understand the effect of the CDSS on team performance and decision making under more realistic circumstances.

## Conclusion

In the ED, where decisions are made under the pressure of time and uncertainty, it is crucial to understand how any proposed CDSS considered for this space effects clinicians' decision making. Our proposed CDSS was intended to support ED physicians' *sensemaking, anticipation,* and *management of complexity* when managing acutely ill paediatric patients with CHD. In evaluating the effect of this CDSS on ED physicians' understanding of CHD and decision-making pertaining to its acute management, improvements in clinician's sensemaking and treatment decision making were noticed. This included an improvement in understanding the CHD anatomy and its effect on how the blood flows through their unique heart and lungs, the influence of the anatomy on normal baseline physiological vital signs and certain limited vulnerabilities these children had because of it. The CDSS also did not distract clinicians away from considering other crucial non-cardiac diagnoses (such as sepsis) and interventions (such as early administration of antibiotics). However, this prototype did not improve CHD specific diagnostic accuracy or rationalization of diagnostic and treatment choices as it did not improve clinicians' anticipation. While this CDSS was successful at supporting clinicians' decision making as it related to determining appropriate interventions, future iteration of this design should focus on improving anticipation among clinicians. Ideally, these sociotechnical solutions should be part of a broader, multifaceted solution that include education and access to additional expertise, to improve the care of these vulnerable patients.

## Data Availability

The raw data supporting the conclusions of this article will be made available by the authors, without undue reservation.
